# An Overview of Pediatric Pulmonary Complications During COVID‐19 Pandemic: A Lesson for Future

**DOI:** 10.1002/iid3.70049

**Published:** 2024-11-07

**Authors:** Zahra Roshanzamir, Fatemeh Mohammadi, Amirhossein Yadegar, Ali Mohammadi Naeini, Katayoon Hojabri, Rohola Shirzadi

**Affiliations:** ^1^ Pediatric Respiratory and Sleep Medicine Research Center Shiraz University of Medical Sciences Shiraz Iran; ^2^ Pediatric Respiratory and Sleep Medicine Research Center, Children's Medical Center, Tehran University of Medical Sciences Tehran Iran; ^3^ School of Medicine Qazvin University of Medical Sciences Qazvin Iran; ^4^ Pediatric Intensive Care Unit, Shiraz University of Medical Sciences Shiraz Iran

**Keywords:** corona virus, COVID‐19, long COVID, pandemic, pediatrics, pulmonary, respiratory complications, SARS‐CoV‐2, viral infection

## Abstract

**Background:**

The pediatric community is considered a suitable target for controlling the spread and mortality of viral diseases. In late December 2019, a respiratory disease due to the novel coronavirus, later COVID‐19, hit the globe. The COVID‐19 global disruption had direct and indirect impacts on different aspects of child health. Therefore, surveillance, preventive approaches, and treatment plans for children came into the spotlight.

**Objective:**

This study aims to discuss the clinical pictures as well as laboratory and radiological findings of the infected children during the COVID‐19 pandemic. The focus of this study is to express the clinical manifestations of respiratory disease in pediatric SARS‐CoV‐2, available therapeutic options, vaccine recommendations, and long COVID sequelae in affected children. This review could serve as a hint for upcoming challenges in pediatric care during future pandemics.

**Results:**

The clinical presentation of COVID‐19 in pediatrics can range from mild pulmonary disease to acute respiratory distress syndrome (ARDS). Supportive care is a crucial component of the management of pediatric COVID‐19. However, the importance of specializing in how to treat patients with more severe conditions cannot be overstated. Additionally, clinicians must consider prevention strategies as well as potential complications.

**Conclusion:**

Although the infected patients are dipping day by day, there is a lack of clinical guidelines for pediatric SARS‐CoV‐2‐associated pulmonary diseases. Understanding of the physicians about all aspects of pediatric care during the COVID‐19 pandemic could lead to enhanced quality of future patient care and safety, reduced costs of health policies, and surveil the risk that patients with respiratory viruses can expose to society.

## Graphical abstract

The focus of this study was to express the clinical manifestations of respiratory disease in pediatric SARS‐CoV‐2, available therapeutic options, vaccine recommendations, and long COVID sequelae in affected children. This review could serve as a hint for upcoming challenges in pediatric care during future pandemics.

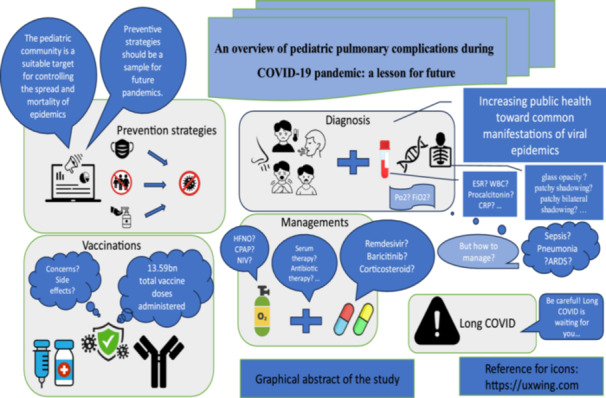

**Abbreviations**
(Ang II): angiotensin II
(ARDS): acute respiratory distress syndrome
(ABG): arterial blood gas analysis
(BiPAP): bilevel positive airway pressure
(BMI): body mass index
(COVID‐19): Coronavirus disease of 2019
(CRP): C‐reactive protein
(CT): chest computed tomography
(CPAP): continuous positive airway pressure
(CXR): chest x‐ray
(CBC): complete blood count
(CK‐MB): creatine kinase‐MB
(E) protein: envelope
(EUA): Emergency Use Authorization
(ESR): erythrocyte sedimentation rate
(ECMO): extracorporeal membrane oxygenation
(FiO2): fraction of inspired oxygen
(FDA): Food and Drug Administration
(GSDMD): gasdermin D
(HFNO): High‐flow nasal oxygen
(HFNC): high‐flow nasal catheter oxygen
(IL): interleukin
(IVIG): intravenous immunoglobulins
(LDH): lactate dehydrogenase
(M) protein: membrane
(MASP‐2): mannose‐binding lectin‐associated serine protease 2
(MIS‐C): multisystem inflammatory syndrome in children
(MERS): Middle East respiratory syndrome
(N) protein: nucleocapsid
(nsp): non‐structural proteins
(NLRP3): NLR family pyrin domain containing 3
(NIV): Non‐invasive ventilation
(NIH): National Institutes of Health
(NG) tube): nasogastric tube
(OI): Oxygenation Index
(OSI): oxygen saturation index
(PFTs): pulmonary function tests
(PCC): post COVID‐19 condition
(PRR): pattern‐recognition receptor
(Paw): mean airway pressure
(PEEP): positive end‐expiratory pressure
(P/F) ratio: PaO2/FiO2
(PBW): predicted body weight
(RSV): respiratory syncytial virus
(RT‐PCR): real‐time polymerase chain reaction
(RNA): ribonucleic acid
(PaO2): arterial oxygen pressure
(RBD): receptor‐binding domain
(S) protein: spike
(SARS): severe acute respiratory syndrome
(SpO2): pulse oximetric saturation
(TLR3): toll‐like receptor 3
(WBC): White blood cell
(WHO): World Health Organization

## 1 Introduction

Since coronavirus disease (COVID‐19) has so far claimed the lives of individuals around the world, it is considered the major public health threat of the century [1]. The virus initially emerged in Wuhan, China, in December 2019 but quickly spread human to human all over the world [2]. As a result, the World Health Organization (WHO) officially announced the launch of a pandemic in March 2020 [3]. During 2020 and 2021, the trends of respiratory viral infections, such as respiratory syncytial virus (RSV), were disturbed by the COVID‐19 pandemic [4]. The coronavirus is of the subfamily Orthocoronavirinae, family Coronaviridae, ealm Riboviria and order Nidovirales [5, 6]. Based on WHO‐released data, as of January 21, 2024, a total of 774,395,593 COVID‐19 cases with 7,023,271 deaths were confirmed (https://covid19.who.int/).

An astounding volume of data has been generated to clarify the clinical findings of COVID‐19 in adults [7]. Potential therapeutic options were discussed earlier. Evidence is dedicated to the potential roles of medications, such as antibiotics, antimalarials, antiparasitic, corticosteroids, anticoagulants, antivirals, and antibody‐based treatments in affected adults. However, pediatric‐specific treatment hints are still considered a challenging topic [8]. Delayed sequelae are also another concern in the younger population [9]. Updated articles on the clinical presentations and treatment approaches for COVID‐19 in children still remain limited [10]. It is crucial to consider the ongoing landscape of the COVID‐19 pandemic concerning its clinical pictures and therapeutic aspects. This poses substantial challenges to clinicians in both realms of diagnosing and formulating a management plan. Moreover, there were additional concerns for the pediatric healthcare system as a result of the overlapping epidemics of influenza, RSV, and SARS‐CoV‐2 [11]. This review study tried to summarize the current knowledge regarding the clinical symptoms and diagnostic and therapeutic options of COVID‐19 in children. As in the past many respiratory diseases became pandemics and took many lives, the world will not be spared from respiratory pandemics in the future. Therefore, insights from the experiences of recent pandemics can rescue future generations in the next pandemic. This review could hint at better decisions for respiratory complications in pediatric patients.

## 2 Method

The search strategy of the current study was as follows: electronic databases, including PubMed and Google Scholar, were searched for the following key terms: (pulmonary pediatric coronavirus‐19) AND (respiratory pediatric COVID‐19) AND (pediatric ARDS) AND (pediatric acute respiratory distress syndrome) AND (MIS‐C) AND (multisystem inflammatory syndrome in children).

### 2.1 Epidemiology

As of May 11, 2023, the cumulative number of pediatric patients diagnosed with COVID‐19 was nearly 15,594,079 cases in the United States, accounting for 17.9% of all cases with an overall rate of 20,718 subjects per 100,000 children [12]. The accurate burden of COVID‐19 child infections at that time is likely underestimated due to a large number of asymptomatic children and those with milder symptoms which could result in lesser rates of pediatric testing [10]. As the omicron variant spread, a rise in the relative proportion of children with positive tests was detected [13]. As a result of inadequate vaccination recommendations, children and adolescents constitute a growing proportion of unvaccinated society and, consequently, the target population for the emerging variants [14].

As of December 2023, nearly 0.4% of the 4.4 million COVID‐19‐related deaths, over 17,400, have contributed to under‐20‐year‐old individuals worldwide. As many as 53% of the 17,400 reported deaths took place among 10‐ to 19‐year‐old adolescents [15]. Ages less than 2 years or 12–19 years, ethnicity, and the existence of comorbidities, were considered determinants of pediatric mortality risk [14].

A study on 19 European countries revealed that COVID‐19‐associated mortality of children aged 15–19 had the highest rates in Malta and Montenegro. However, death rates for this age group were very low in countries such as Iceland and Slovenia [16]. As compared with high‐income countries, low‐ and middle‐income countries had higher rates of pediatric COVID‐19‐associated mortality. One reason could be attributed to the resource limitations [17, 18, 19].

### 2.2 Transmission

The main source of infection is the person contaminated with the virus via respiratory droplets as well as aerosol spread [20]. Moreover, another possible cycle is the maternal‐to‐infant transmission through the placenta, delivery, or breastfeeding [21]. COVID‐19 transmission could be affected by the type of viral source manifestations, exposure characteristics, SARS‐CoV‐2 variant, and the load of the virus [14]. The infected subject may not have clinical symptoms [17, 22, 23]. Another source of infection is those who are in their incubation period. In the case of children, a family member is considered to be the viral source in most cases (more than 80%) [24]. Infected children could also transmit the disease to their family members [25]. Studies show that the load of the virus in the nasopharynx of children is similar to or even higher than the virus load in the adult's nasopharynx. Therefore, children have the ability to transmit the disease to other children and adults [26, 27]. However, research showed higher rates of Delta variant transmission were dedicated to adults than children, especially among households [20]. Accordingly, the likelihood of transmission rates within children and adolescents has been rising with the introduction of highly transmissible variants [14].

### 2.3 Pathophysiology

SARS‐CoV‐2, as an RNA virus, has a positive sense and single‐stranded RNA with a genome size of 29.9 kb. It has structural proteins, including nucleocapsid (N) protein, envelope (E) protein, spike (S) protein, membrane (M) protein, and 16 nonstructural proteins (nsp1‐16) [28]. The SARS‐CoV‐2 is capable of entering cells through interactions between the receptor‐binding domain (RBD) located within the viral S1 subunit and the angiotensin‐converting enzyme 2 (ACE2), which functions as the host cell receptor [29]. In the presence of ACE2, which functions as a carboxypeptidase, angiotensin 1–7 is produced from angiotensin II (Ang II). The interaction between ACE2 and the viral S protein can result in reduced ACE2 function, leading to enhanced Ang II signaling and pro‐inflammatory/pro‐thrombotic pathways. Endothelial damage can arise from dysregulated RAAS through oxidative stress. Ang II‐induced elevated reactive oxygen species along with decreased nitric oxide secondary to low angiotensin 1–7 levels can negatively affect endothelial function. As a result of endothelial dysfunction, prothrombotic factors like P‐selectins, angiopoietin‐2, and endothelin‐1 can be generated in the endothelium. In comparison with other Coronaviruses, SARS‐CoV‐2 exhibits a higher affinity for the ACE2 receptor [30]. Innate immunity provides early defenses against SARS‐CoV‐2 [29, 30]. A pattern‐recognition receptor (PRR), toll‐like receptor 3 (TLR3), can identify SARS‐CoV‐2 RNA once it enters the cell, leading to the induction of NLR family pyrin domain containing 3 (NLRP3) gene transcription [29]. Upon interaction between SARS‐CoV‐2 nucleocapsid protein and mannose‐binding lectin‐associated serine protease 2 (MASP‐2), found in the microvasculature, the complement is overactivated. This results in platelet stimulation and overexpression of pro‐inflammatory cytokines from endothelial cells as well as endothelial and monocyte tissue factors [30]. The severe inflammatory response caused by COVID‐19 can cause organ failure and even death through the release of cytokines, such as interleukin‐18 (IL‐18), IL‐1β, and gasdermin D (GSDMD) [29, 31]. CD4+T cells show higher antiviral effects against COVID‐19 compared to CD8+T cells. CD4+T cells are specific for the overexpressed spike, M, and nucleocapsid antigen as well as nsp3, nsp4, and ORF8 [29].

### 2.4 Clinical Symptoms and Complications

The clinical symptoms in children at the time of the outbreak of middle east respiratory syndrome (MERS) and severe acute respiratory syndrome (SARS) were milder than in adults. Similarly to the previous Corona types, COVID‐19 manifests milder symptoms in children [32]. Clinical symptoms often emerge after a 2‐to‐7‐day incubation period. Previous studies declared that manifestations in terms of prevalence include fever (47.5%), cough (41.5%), diarrhea (8.1%), nausea/vomiting (7.1%), fatigue (4%), and sore throat (2%) [33]. Evidence showed that the duration of fever was 3 days on average, with a temperature of more than 39 degrees Celsius in (8.8%) of cases [33].

In general, asymptomatic pediatric cases comprise 15.8%–65% of the total [10]. Cutaneous manifestations, such as maculopapular, urticarial, and vesicular rashes along with painful lesions, known as COVID toes, could lead to the suspicion of coronavirus infection. Rhinorrhea is another low‐incidence finding (10%–22%) of COVID‐19 in children. Anosmia is regarded as a rare symptom but a strong predictor of a COVID‐19 positive test. Croup could also be considered a presentation of the Omicron variant and associated with an increased likelihood of a positive test [10].

Upper respiratory manifestations were reported in 19.3% of patients, with pneumonic symptoms being the most common (64.9%) complication [33]. Pneumonia is also classified according to its severity:

1. Mild pneumonia: absence of severe symptoms and presence of tachypnea with respiratory rates of ≥ 60, ≥ 50, and ≥ 40 in pediatrics aged less than 2 months, 2–11 months, and 1–5 years, respectively.
2. Severe pneumonia: Respiratory symptoms, such as cough or shortness of breath with at least one of the following conditions:

- SpO_2_ < 90.
- Very severe respiratory distress implicated by chest indrawing and grunting.
- Danger symptoms, defined as inability to breastfeed, lethargy, and decreased level of consciousness [34, 35, 36, 37, 38].

Infants under 1 year old have an increased likelihood of having severe symptoms compared to other age groups of children. In general, the progression to severe forms of symptoms was approximately 10% in under 1‐year infants compared to 7.3% in 1–5, followed by 4.2% in 6–10, and 4.1% in 11–15 years of age [39, 40].

3. Acute respiratory distress syndrome (ARDS): the parameters for ARDS diagnosis in an affected child are as follows [41].

- Having the onset of respiratory symptoms during the first 2 weeks of the disease.
- Presence of bilateral involvement in chest x‐ray (CXR) or chest computed tomography (CT) scan.
- Having at least positive end‐expiratory pressure (PEEP) equal to five, by continuous positive airway pressure (CPAP) or bilevel positive airway pressure (BiPAP) or mechanical ventilation, with oxygenation, as shown in Table 1.

**Table 1:** Measurement of oxygenation in pediatric ARDS.

Type of ventilation	Oxygenation
Bilevel NIV or CPAP ≥ 5 cm	PaO_2_/FiO_2_ ≤ 300 mmHg or SpO_2_/FiO_2_ ≤ 264
Invasive mechanical ventilation	Mild: 4 ≤ OI < 8, 5 ≤ OSI < 7.5
	Moderate: 8 ≤ OI < 16, 7.5 ≤ OSI < 12.3
	Sever: OI ≥ 16, OSI ≥ 12.3

*Note:* OI (Oxygenation Index): FiO_2_ × mean airway pressure (paw) × 100/PaO_2_, OSI (Oxygenation Saturation Index): FiO_2_ × Paw × 100/SpO_2_.

ARDS represents a heterogeneous syndrome that encompasses a variety of etiologies, such as shock, trauma, aspiration pneumonia, viral or bacterial pneumonia, sepsis, and pancreatitis [42, 43]. As part of the pathophysiology of classical ARDS, fibrin‐rich exudates, called hyaline membranes, are formed secondary to the dysregulation of coagulation and fibrinolysis, which interferes with the air–blood barrier activity. The appearance of an intra‐alveolar hyaline membrane results in fibroblast proliferation, edema, and the expansion of the interstitial cavity. Lung edema secondary to ARDS results in a decrease in lung volume and an increase in shunt fraction, which contributes to hypoxia. As a result of a reduced lung volume, the respiratory system is less compliant [42]. There are several distinctions between COVID‐19‐related ARDS and classical ARDS [44, 45]. Patients with ARDS associated with COVID‐19 may have slightly higher respiratory system compliance following intubation compared to classical ARDS in the case of equivalent arterial oxygen pressure (PaO_2_)/fraction of inspired oxygen (FiO_2_) ratio [42]. COVID‐19‐associated ARDS is driven by endothelial inflammation and vascular thrombosis as well as pulmonary fibrosis and infiltration, resulting in altered alveolar homeostasis [46]. Activated endothelial cells promote rolling, adhesion, and migration of inflammatory cells, as well as inflammatory signaling and coagulation, leading to barrier disruption, diffusion impairment, and coagulation initiation [43]. So far, several studies have revealed a greater likelihood of thrombotic events in individuals with COVID‐19‐induced ARDS than those with other etiologies of ARDS [42, 47, 48, 49]. COVID‐19‐associated vascular abnormalities, such as endothelial cell damage, microthrombosis, and hyperplasia of pulmonary capillaries can result in a different pathophysiology and treatment response in comparison to classical ARDS [43]. Therefore, a vasocentric pathophysiology implies a potential application for vascular interventions like therapeutic anticoagulation [50].

In classical ARDS, hypoxia is primarily caused by atelectasis and consolidation, leading to a rise in physiological shunt fractions [50]. However, hypoxemia in COVID‐19‐induced ARDS may result from dead space expansion and simultaneous intrapulmonary shunt secondary to hypoxic pulmonary vasoconstriction impairment, imbalance of lung perfusion, and pulmonary vascular microthrombus [43]. Unlike classical ARDS, COVID‐19‐related ARDS with severe hypoxemia, defined as having oxygen saturation of less than 70% and partial pressure of arterial oxygen of less than 40 mmHg, can present without dyspnea. This observation is referred to as “happy hypoxemia” or “silent hypoxemia,” which can be explained by potential mechanisms, such as poor response to hypoxia or cardiorespiratory compensation to hypoxemia [43]. According to the Berlin Criteria, classical ARDS should occur within 7 days of establishing a clinical condition or respiratory complications; however, COVID‐19‐induced ARDS can take longer to develop [42, 46, 51, 52, 53]. In comparison to classical ARDS, COVID‐19‐related ARDS is associated with a prolonged need for mechanical ventilation [42]. A higher mortality rate was mentioned in COVID‐19 cases with ARDS who have decreased compliance of the respiratory system as well as increased levels of D‐dimer [45]. An increased risk of in‐hospital mortality among pulmonary septic shock cases with severe COVID‐19‐associated ARDS compared to classical ARDS was also reported [54].

Hypoxia is a well‐known complication of COVID‐19. Therefore, the following points should be considered in the oxygenation measurements of COVID‐19 patients with respiratory syndromes:

The Oxygenation Index (OI) should be considered the first criterion for assessing the severity of pulmonary involvement in patients under mechanical ventilation [55, 56]. It was initially studied as a prognostic factor for acute hypoxemic respiratory failure in children [57]. OI is calculated using the following equation: OI = (FiO_2_ × mean airway pressure × 100)/PaO_2_ [58]. PaO_2_/FiO2 (P/F) ratio should be evaluated for diagnosis of ARDS in candidates for O_2_ therapy with at least a PEEP of 5 cmH_2_O, by CPAP or BiPAP [59]. Pulse oximetry is helpful in children who are unsuitable for invasive methods like arterial blood gas analysis (ABG) [60]. As a result, evaluation of oxygen saturation index (OSI), computed as (FiO_2_ × MAP × 100)/SpO_2_, instead of OI in patients under mechanical ventilation can be predictive of the severity of pulmonary involvement [61, 62]. Accordingly, the pulse oximetric saturation (SpO_2_)/FiO_2_ should be analyzed instead of the P/F ratio in the candidates for O2 therapy with at least PEEP equal to five with noninvasive ventilation (NIV), CPAP or BiPAP. The goal of O_2_ therapy in the mentioned group is to keep SpO_2_ in the range of 88%–97% [59, 61, 63].

Several weeks to months after COVID‐19 infection, children and adolescents are particularly susceptible to postinfectious hyperinflammatory syndromes, known as multisystem inflammatory syndrome in children (MIS‐C) [30, 64]. In April 2020, COVID‐19‐associated MIS‐C was first reported in eight children with positive antibody tests who developed fever, cardiovascular complications, and generalized inflammation [64, 65]. It is described as the inflammation of organs, such as the heart, lungs, kidney, brain, skin, eye, and gastrointestinal tract [64, 66]. Symptoms, such as fever, fatigue, skin rash, diarrhea, vomiting, conjunctivitis, cheilitis, extremity changes, and abdominal pain as well as increased serum inflammatory markers could raise suspicion for MIS‐C diagnosis [64, 66]. Persistent fever for at least 24–48 h is considered a mandatory criterion for MIS‐C diagnosis in children [67]. Approximately 90% of MIS‐C patients suffer from gastrointestinal complaints [68]. The cardiovascular system is involved in more than 80% of MIS‐C cases [67]. An estimated one‐third to one‐half of patients with MIS‐C experience respiratory symptoms [68]. These symptoms can vary from mild tachypnea and hypoxemia to respiratory failure with pulmonary infiltrates. However, severe, acute COVID‐19 is more likely to develop lower respiratory symptoms compared to MIS‐C [67]. Acute kidney injury, coagulopathy, myocardial damage, myocarditis, coronary artery aneurysms, and respiratory distress were reported among its complications [66, 69].

Individuals with MIS‐C have a higher likelihood of developing thrombosis [30, 70]. Among children with MIS‐C, thrombosis was reported to be associated with elevated D‐dimer, thrombocytopenia, ICU hospitalization, and noncardiogenic pulmonary edema [71]. An estimated 15.8% of COVID‐19‐positive adults suffer from pulmonary embolism, which is potentially life‐threatening [72]. The incidence of pulmonary embolism in children was reported to be rare, with two to six cases per 10,000 patients discharged from hospitals [73]. Children are less likely to suffer from pulmonary embolisms than adults. However, more frequent pulmonary embolisms were reported in children hospitalized due to COVID‐19 [74].

### 2.5 Laboratory Findings

The findings of the complete blood count (CBC) test in children with SARS‐CoV‐2 variants resemble other respiratory viral diseases. White blood cell (WBC) count is relatively normal. Leukopenia was detected in only 38% of cases. Lymphopenia, which is one of the findings of the adult index, has been seen in only 20% of cases and is not a clear laboratory finding in children [75]. Thrombocytopenia is a hallmark in 14% of infected children [76]. Procalcitonin levels are also in range in most cases, whereas high levels could indicate a bacterial superinfection [34]. Elevated c‐reactive protein (CRP) concentrations could be found in 18% of the infected cases [34]. An increment in serum concentrations of inflammatory biomarkers, such as CRP and procalcitonin, transaminitis, coagulopathy indicators like D‐dimer, creatine kinase‐MB (CK‐MB), and lactate dehydrogenase (LDH) is expected in systemic involvements of SARS‐CoV‐2 [10]. Clinicians should raise their suspicion of pulmonary involvements in pediatrics with decreased levels of thrombocytes and lymphocytes, or increased values of viral load, ESR, procalcitonin, and CRP [9]. Literature also showed that IL‐10, IL‐6, and interferon‐gamma levels increased in 75%, 37%, and 25% of severe infections, respectively [10]. In general, interstitial inflammation and extensive alveolar edema, macrophage recruitment in the alveolar tissue, mononuclear cell deposition, and release of inflammatory molecules can predispose the respiratory system to develop pulmonary damage [77].

### 2.6 Radiological Findings

Findings, such as predominant involvement of the right subpleural region, small patchy infiltration lesions, and interstitial changes in CXR, would prompt physicians to consider a suspicion of SARS‐CoV‐2 [53].

Common chest CT scan findings include ground glass opacity (67%), patchy shadowing (37%), and patchy bilateral shadowing (21%). In 49% of cases, the longitudinal axis was parallel to pleural lesions. The lung lesions in chest CT were shown to be subpleural in 95% of infected children with lung involvement. The mentioned lesions appear to occur in the early stages of the disease. Bilateral involvement is rare in children. Studies have not reported lymphadenopathy in any of the ill pediatric subjects. Moreover, one case was described with pleural effusion [75]. Chest CT typically shows bilateral, peripheral ground glass patterns, which overlap with other diagnoses. Therefore, chest CT is considered to have a high sensitivity and relatively low specificity in COVID‐19 diagnosis. The American College of Radiology does not recommend a CT scan for screening or as the first diagnostic test for COVID‐19 [78].

### 2.7 Diagnostic Criteria

Physicians should be alert for SARS‐CoV‐2 diagnosis when a child has acute respiratory symptoms, such as fever, cough, and sore throat during the last 10 days and needs hospitalization, as well as one of the following epidemiological criteria:

1. Traveling or staying in epidemiological areas for corona has been declared a pandemic in the current situation.
2. Close contact, as defined by living at the same home, being a classmate, or traveling in any vehicle, with a suspected or confirmed coronavirus‐infected person in the last 14 days.
3. The presence of any suspicious or definite infected person in the family, considering that 80% of infected children had infected family members. This criterion is more important than others [79].

All suspicious cases should be examined for a definitive diagnosis. The definitive diagnosis of COVID‐19 is made using a nucleic acid test performed by real‐time polymerase chain reaction (RT‐PCR) from a throat swab, sputum, stool, or blood sample. Virus ribonucleic acid (RNA) is better detected in nasopharyngeal specimens than in the throat. Lower respiratory specimens are more diagnostic compared to upper respiratory specimens. The presence of viral findings in the blood could indicate the probability of a severe illness [80]. It is of great value to consider differential diagnoses in children undergoing diagnosis of COVID‐19. If symptoms of fever, cough, rhinorrhea, and sore throat or pneumonia are present in patients, COVID‐19, as well as influenza A and B, should be among the first in the differential diagnosis list. Therefore, it is recommended to assess a throat swab sample for influenza PCR types A and B in a child who needs to be hospitalized with the possibility of COVID‐19. If flu is detected, it should be treated in time. Various methods, such as multiplex RT‐PCR or triple antigen tests have been introduced to assess the presence of influenza A and B, RSV, or SARS‐CoV‐2 in affected individuals [11]. Another differential diagnosis of COVID‐19 is bacterial pneumonia, often associated with a high fever. Consolidations in CXR, leukocytosis with neutrophil predominance as well as increased erythrocyte sedimentation rate (ESR), and CRP provide diagnostic clues for bacterial pneumonia [81].

### 2.8 Risk Factors of Severe Disease

**Figure 1:**
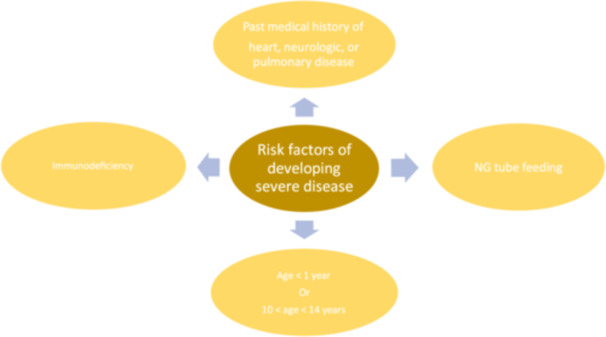
Risk factors of developing severe COVID‐19 infection in pediatrics. Depicts the factors, which increase the likelihood of developing severe SARS‐CoV‐2 infections in children. NG tube, Nasogastric tube.

The risk factors of severe SARS‐CoV‐2 development are as follows: (Figure 1.)

- A positive past medical history of cardiological, neurological, or pulmonary disease.
- Nasogastric tube (NG tube) feeding.
- Immunodeficiency, both primary or acquired.
- Age less than 1 year or 10–14 years of age.

### 2.9 Management and Treatment

At home, it is recommended to follow the isolation tips. Antipyretics, such as acetaminophen or ibuprofen are suggested to control fever. Another recommendation is to consume enough calories and fluids. Warning signs, such as not breastfeeding, increased breathing rate, drowsiness, and shortness of breath offer the parents to immediately bring their children to the hospital. Moreover, another visit in the next 48 h is suggested [81, 82].

The presence of ARDS, severe pneumonia, or symptoms of sepsis or dehydration could serve as a hint to hospitalize the pediatric patient. Oxygen therapy is the first step in managing children with any of the symptoms, including tachypnea according to age range, granting, subcostal and suprasternal retraction, central cyanosis, shock, seizure, and drowsiness. During resuscitation, the oxygen therapy should maintain the SpO_2_ higher than 94%, and after that, the SpO_2_ should be kept above 90%. All care centers for children with respiratory viral infections should have pulse oximeters, sterile and disposable oxygen systems, and oxygen transfer devices, such as simple face masks and masks with reservoir bags [83, 84]. Intravenous fluid therapy should be used cautiously in the lack of dehydration symptoms because over‐fluid therapy could interfere with oxygenation. Empiric antibiotic treatment in children with sepsis symptoms should begin within the first hours of hospitalization. Empiric therapy with a neuraminidase inhibitor should also be initiated in case of suspected influenza‐contacted cases until the flu PCR result becomes available [52, 61, 84].

### 2.10 Management of MIS‐C

There are very few cases of MIS‐C, but it can have life‐threatening consequences [85]. It is estimated that approximately two‐thirds of patients diagnosed with MIS‐C are required to be admitted to an intensive care unit [86]. Immunomodulatory drugs like intravenous immunoglobulins (IVIG) and glucocorticoids, such as methylprednisolone or prednisone recommended as the first‐line treatment by current guidelines [66, 69, 85]. Intravenous corticosteroids are initially administered, followed by oral administration and a gradual tapering off [68]. A more aggressive treatment approach is recommended in the initial stages of treating critically ill patients to alleviate cardiovascular complications faster [68]. The critically ill patients may require mechanical ventilation, extracorporeal membrane oxygenation (ECMO), and inotropic agents [85, 87]. Anticoagulation is recommended in those with laboratory tests indicating a hypercoagulable state for possible thrombosis development [68].

### 2.11 Management of ARDS

Since ARDS has a variety of etiologies, no uniform approach can be considered. However, COVID‐19‐associated ARDS may benefit from antiviral therapy, anti‐inflammatory medications, and anticoagulation, depending on the pathogenesis of COVID‐19. It is recommended to administer high‐flow nasal catheter oxygen (HFNC) inhalation to COVID‐19 cases with acute hypoxemic respiratory failure resistant to conventional oxygen therapy to sustain their peripheral oxygen saturation of 92‐96% [43]. While COVID‐19‐related ARDS may have a different pathogenesis from classical ARDS, patients with COVID‐19 are advised to follow the respiratory support protocol used in classical ARDS. Among these techniques are low tidal volume ventilation (4–8 mL/kg of predicted body weight) and preserving plateau pressure below 30 cm H_2_O. Mechanically ventilated subjects with moderate to severe COVID‐19‐related ARDS and hypoxemia resistant to optimal ventilation may benefit from VV‐ ECMO, neuromuscular blockers, increased PEEP levels, recruitment maneuvers (incremental PEEP), or prone ventilation for 12–16 h. It is crucial to provide individualized care for individuals with COVID‐19 who do not exhibit typical ARDS [43]. Before the SARS‐CoV‐2 pandemic, high‐flow nasal oxygen (HFNO) was not considered a prevailing respiratory support modality. High amounts of humidified oxygen could be supplied by HFNO [88]. HFNO system can transfer up to 60 l of oxygen per minute and produce FiO_2_ with an efficiency of almost 100%. However, the HFNO transmission system can only carry up to 15 l of oxygen per minute in children. The adult‐specified transmission system should be replaced in case of higher demands in children. HFNO should not be used if hemodynamic instability and hypercapnia are present [89]. NIV and CPAP are the other means of respiratory support with the application of bi‐level and single levels of positive pressure, respectively [88]. A negative pressure room is required for the NIV applicant. As a result, hemodynamic instability and multiorgan failure are considered the two contraindications of NIV use. Evidence has not supported the use of NIV in influenza pandemics due to the increasing possibility of delayed intubation. Accordingly, intubation experts are recommended to monitor the NIV‐candidate children with SARS‐CoV‐2 for several hours to check the trial's tolerance and estimate the patient's condition [90, 91]. Intubation should be performed immediately in case of not tolerating or contraindications. Due to the oxygen loss during the intubation, it is recommended to supply oxygen with 100% FiO_2_ for 5 min using NIV, HFNO, or a mask before the intubation [92]. Literature showed recent NIV devices do not lead to an increased risk of disease transmission [91].

### 2.12 Ventilatory Support

#### 2.12.1 Mode of Ventilation

Both control mode and assist mode can be set depending on the patient's condition [41, 93].

#### 2.12.2 Tidal Volume

It is suggested to apply a lower‐tidal‐volume strategy compared to the physiological range of the body. The recommended approach starts with 6 cc/kg predicted body weight (PBW) and could level up to 8 cc/kg PBW if necessary [41, 93].

#### 2.12.3 PEEP

Relatively high levels of PEEP (10–15 cmH_2_O) are gradually titrated according to the patient's oxygenation rate and hemodynamics. A PEEP above 15 cmH_2_O may be required for severe ARDS, although efforts are being made to limit plateau pressure. PEEP remains at least 10 cm H_2_O for ARDS application. Lower optimal SpO_2_ levels (92%–88%) may be better provided for PEEP candidates [41].

Permissive hypercapnia, with a PH range of 7.30–7.15, should be considered for patients with moderate to severe ARDS to reduce ventilator injuries.

Sedation could also result in increased tolerance of mechanical ventilation, enhanced oxygen supply, and decreased respiratory work [41].

Neuromuscular blocker drugs could be served in cases with remaining intolerance to the ventilator despite sedation consumption [94].

**Figure 2:**
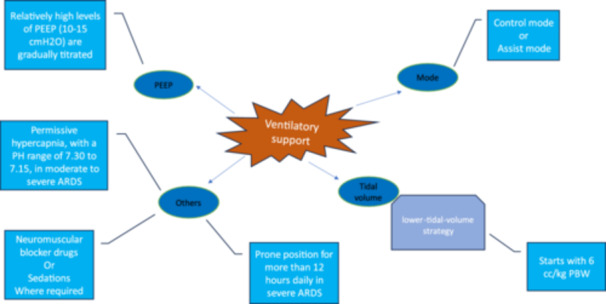
Ventilatory support settings in children with ARDS in COVID‐19 pandemics. The basics of ventilatory support settings for patients with ARDS. The mode, tidal volume, PEEP, and additional settings are mentioned. ARDS, acute respiratory distress syndrome; PBW, predicted body weight; PEEP, positive end‐expiratory pressure.

Prone position: In children with severe ARDS, the prone position is recommended for more than 12 h daily. Furthermore, trained and experienced personnel are required for advanced proning response [95] (Figure 2).

### 2.13 Drug Therapy

#### 2.13.1 Remdesivir

It is an analog nucleotide drug that binds to the virus polymerase and inhibits virus replication. It has been shown to be effective in in‐vitro studies in SARS‐CoV‐2 with severe respiratory disease. The US Food and Drug Administration (FDA) has published an Emergency Use Authorization (EUA) to use remdesivir for pediatric patients suspected of or confirmed COVID‐19. The dose was used as a loading dose of 5 mg/kg on the first day and then 2.5 mg/kg daily from the second to the fifth day. In children weighting over 40 kg, the dose used is the same as for adults, and the dose is 200 mg on the first day and 100 mg daily from the second to the fifth day. The recommended duration of remdesivir in pediatric patients is 5 days if not under invasive mechanical ventilation and 10 days if under invasive mechanical ventilation. It is important to notice that only the lyophilized powder form of this drug should be used in pediatric patients. Serial creatinine and liver enzymes should be assessed before and during treatment [96].

#### 2.13.2 Baricitinib

The second drug that was approved by the FDA is baricitinib. Baricitinib is a Janus kinase inhibitor that is used in Arthritis Rheumatoid treatment. The other mechanism of this drug is interfering with the entry of the virus into the cell. This mechanism can be useful in COVID‐19 treatment.

#### 2.13.3 Corticosteroid

National Institutes of Health (NIH) recommended using dexamethasone only in children who need NIV, HFNO, or mechanical ventilation due to COVID‐19 pneumonia. It is not suggested in patients for whom O_2_ therapy without NIV or mechanical ventilation is set [97].

Treatment plans for children hospitalized with COVID‐19 are as follows:

1. Anti‐coagulants are recommended as a prophylactic option in those older than 12 with no counterindications.
2. Remdesivir is considered in hospitalized children aged 12–17 years with an increased likelihood of developing severe lung disease, such as children with immunodeficiency despite COVID‐19 vaccination, who do not require oxygen therapy or improve with conventional oxygen therapy.
3. Intubated children on mechanical ventilation or patients on ECMO benefit from dexamethasone. However, previous literature has shown no mortality reductions by receiving remdesivir in these cases. Individuals with increasing oxygen demand are shown to be alleviated with remdesivir plus dexamethasone. Moreover, children who need high flow or NIV oxygen therapy are recommended to be prescribed dexamethasone or remdesivir plus dexamethasone.
4. Tocilizumab or baricitinib had efficacy in studies of hospitalized children older than 2 years who received dexamethasone with no improvement in SpO_2_ levels [98].

Remdesivir has also shown to be helpful in the Omicron variant. A 10‐day treatment with remdesivir is suggested in patients with no significant improvement after a 5‐day treatment or in those with immunodeficiency. Moreover, it is recommended to continue taking remdesivir in children prescribed remdesivir before intubation.

Dexamethasone is advised for patients on ECMO or mechanical ventilation. Furthermore, dexamethasone or remdesivir plus dexamethasone is a beneficial choice in children who need high‐flow or NIV oxygen therapy. However, it is not recommended to use dexamethasone in patients receiving conventional oxygen therapy. Dexamethasone is prescribed for children with COVID‐19 at the advised dosage of 0.15 mg/kg with an upper limit of 6 mg daily for a maximum of 10 days.

### 2.14 Vaccination

Vaccination is considered the cornerstone of an effective way to avoid COVID‐19 and its associated ailments, such as RSV infection [4, 14]. As a result, severe forms of the disease have been reported more in unvaccinated cases than in vaccinated ones. Pediatric COVID‐19 vaccination licensure could be received after testing in adults [14]. A public dilemma regarding vaccination is parental consent in the case of COVID‐19 vaccination. Literature shows that the parent's acceptance rate of vaccination toward their children is approximately 57%. Enhanced awareness of the vaccine, trust in the COVID‐19 vaccine, improved availability and accessibility of vaccination, and government incentives could increase the likelihood of parental willingness toward vaccination. However, psychological distress and worries can be considered contributing factors to being reluctant to pediatric vaccination [99]. It has been shown that vaccination does not result in growth and developmental disturbance [100]. Due to the controversies in the vaccination of under 5 years children, health policymakers should outline the guidelines considering the community benefits of vaccinating based on the geographical region and its epidemiological characteristics [101]. Prior research reported post‐COVID‐19‐vaccination myocarditis and pericarditis. A meta‐analysis revealed that the pooled incidence of myocarditis and pericarditis was 0.00063% and 0.000074%, respectively. The results showed that the male children and adolescents and the second dose of Pfizer were more likely to develop myocarditis and pericarditis. Therefore, children and adolescents with a history of COVID‐19 vaccination and suggestive presentations could offer the clinicians a hint to a possible myocarditis or pericarditis diagnosis [102]. So far, a two‐dose 50 and 25 µg mRNA‐1273 series has been licensed in the United States for ages 6 to 11 years old and 6 months to 5 years old, respectively. Moreover, the United States authorized a two‐dose 10 µg BNT162b2 and a three‐dose 3 µg BNT162b2 primary series for ages 5–11 years old and 6 months to 4 years old, respectively [14]. The Advisory Committee on Immunization Practices also recommended the administration of a monovalent mRNA vaccine booster, referred to as the third dose, after a period of 5 months following the last primary dose in individuals older than 5 years of age. Booster doses offered additional protection in immunocompromised individuals during BA.2/BA.2.12.1 and BA.4/BA.5 [103]. Additional mutations in Omicron BA.2/BA.2.12.1 and BA.4/BA.5 resulted in enhanced resistance to antibodies produced following monovalent booster doses or BA.1 infection [104, 105, 106, 107]. Considering the reduced efficacy of monovalent mRNA vaccines in the context of Omicron variants, the FDA approved bivalent vaccine versions at least 2 months after the primary series were administered [103].

### 2.15 Pediatric Long COVID

In the midst of the COVID‐19 pandemic, literature mostly focused on mental health complications of SARS‐CoV‐2; however, after a short while, thrombotic events, inflammatory states, and microangiopathy also caused challenges for physicians [9]. In a confirmed COVID‐19 case, a long COVID or post‐COVID‐19 condition (PCC) is diagnosed when at least one symptom remains after 12 weeks and cannot be justified by other factors. Excessive fatigue, reduced exercise tolerance, chest discomfort, prolonged cough, and dyspnea, as well as impaired cognition, such as headache, sleep difficulties, and impaired concentration, are among PCC manifestations, which can either persist or evolve following acute infection. These symptoms have the ability to interfere with daily activities and can alter or recur throughout time [108]. Allergic conditions, older age, and higher body mass index (BMI) levels can increase the likelihood of developing persistent symptoms after COVID‐19 infection [108]. The prevalence of PCC in pediatrics has not been well defined and varies between 1.8% and 96.6% [108]. PCC is characterized by a number of pathophysiological changes, including direct endothelial deterioration, regional inflammation, and a thromboembolic environment. Endothelial cells can be directly infected by SARS‐CoV‐2 through ACE2, causing the microangiopathy and fibrotic lesions of affected patients. Microvascular angiography has shown extensive microangiopathy in more than 65 percent of subjects after 3 months of incubation. Clinicians should be alert for ventilation‐perfusion [V/Q] mismatch in recovered pediatrics [9]. Children with pulmonary‐involved long COVID can be monitored through lung ultrasound or pulmonary function tests (PFTs) [108]. Mild obstructive pattern is considered the predominant ailment of pediatric PCC‐associated respiratory complications. Therefore, empirical respiratory inhalers are one of the therapeutic options in children with PFT impairments [109].

## 3 Discussion

The current review aimed to reveal the pediatric respiratory manifestations of SARS‐CoV‐2 and discuss the therapeutic options. Previous systematic reviews were conducted to show the epidemiologic characteristics of COVID‐19 as well as laboratory test results and radiologic findings associated with pediatric COVID‐19 [110, 111, 112, 113]. Similar to the present study, previous publications announced that asymptomatic patients constituted the major population of pediatric Coronavirus‐19. Moreover, fever and cough were the most common findings in the symptomatic individuals [112, 114]. Another study published by Christophers et al. declared that gastrointestinal manifestations as well as mental health problems like depression and anxiety should be also considered in the affected pediatric population [115]. A review conducted by Yasuhara et al. mentioned Ground‐like opacities as the most common associated radiologic findings. In addition, increased levels of D‐dimer and CRP along with lymphopenia were noted as the main laboratory results. Gastrointestinal symptoms, shock, left ventricular systolic dysfunction, and enhanced inflammatory biomarkers were stated as the manifestations of associated COVID‐19 MIS‐C [116]. Another publication on MIS‐C also established that the following presentations may suggest the presence of MIS‐C in children: first, being in shock status along with multiorgan failure, with cardiovascular and gastrointestinal organs being the most affected organs; second, involvement of cutaneous and mucosal tissues along with less symptoms of shock; third, referral with respiratory failure along with positive COVID‐19 PCR results from throat swab [117]. The current study also tried to reveal the associated complications, such as thromboembolism and MIS‐C in pediatric COVID‐19. ARDS, thromboembolism, and MIS‐C are life‐threatening conditions that can be caused by COVID‐induced systemic hyperinflammation, which differentiates SARS‐CoV‐2 from other viruses [68, 118, 119]. Lopez‐Leon et al. mentioned that long‐COVID was diagnosed in 25.24% of the individuals, with mood symptoms constituting 16.50% of findings, followed by fatigue (9.66%), and sleep disorders (8.42%) [120]. The current study also highlighted the clinical presentations of long‐COVID conditions. Investigating the epidemiological characteristics, clinical pictures, associated complications, as well as available therapeutic options for respiratory ailments in the pediatric COVID‐19 population could enhance the clinician's sensitivity toward future possible pandemics. Further studies are required to fill the existing gaps in the COVID‐19 complex behavior in pediatric populations.

## 4 Conclusion

Pulmonary disease in children with COVID‐19 has a clinical range from mild to ARDS. Supportive care is the most crucial point in managing affected pediatrics during the COVID‐19 pandemic. Among the FDA‐approved drugs for treating COVID‐19 in children are remdesivir, baricitinib, and tocilizumab. Dexamethasone is considered to have a critical role in managing pediatrics with NIV or mechanical ventilation, or ECMO. In addition to treating acute infections, healthcare providers must also consider prevention strategies and possible sequelae. It is necessary to prepare clinicians for future pandemics by providing more guidelines to enhance their awareness of the available strategies used during recent pandemics.

## Author Contributions

**Zahra Roshanzamir:** conceptualization, investigation, visualization, writing–review and editing. **Fatemeh Mohammadi:** conceptualization, investigation, visualization, writing–review and editing. **Amirhossein Yadegar:** conceptualization, investigation, visualization, writing–original draft. **Ali Mohammadi Naeini:** conceptualization, investigation, visualization, writing–original draft. **Katayoon Hojabri:** conceptualization, investigation, visualization, writing–original draft. **Rohola Shirzadi:** conceptualization; investigation; supervision; visualization; writing–review & editing.

## Ethics Statement

The authors have nothing to report.

## Conflicts of Interest

The authors declare no conflicts of interest.

## Data Availability

The datasets used and/or analyzed during the current study are available from the corresponding author upon reasonable request.
